# Dataset on subjective well-being (happiness) among university students in Indonesia

**DOI:** 10.1016/j.dib.2026.113079

**Published:** 2026-07-21

**Authors:** Mamang Efendy, Andik Matulessy, Amanda Pasca Rini, Diah Sofiah

**Affiliations:** Universitas 17 Agustus 1945 Surabaya, Surabaya, Indonesia

**Keywords:** Oxford happiness questionnaire, Psychometric validation, Cross-cultural adaptation, Online survey data, Demographic variables, Mental health measurement, Scale reliability, Secondary data analysis

## Abstract

This study is a non-experimental study aimed at collecting data on subjective well-being (happiness) among university students in Indonesia. The study employed non-random sampling using an online questionnaire distribution method via social media platforms such as WhatsApp groups or Instagram. Happiness data were collected using the Oxford Happiness Questionnaire (OHQ) by [1], consisting of 29 items that had undergone adaptation procedures for the Indonesian version in accordance with the adaptation procedures outlined in [2]. A total of 804 university students completed the questionnaire, comprising 276 male students and 528 female students, with ages ranging from 18 to 41 years (mean age = 21.23, SD = 3.73). The research data also includes demographic information such as gender, educational level, and province of origin. This research data on subjective well-being (happiness) among university students in Indonesia can be reused by other researchers for various study or research purposes, such as psychometric analysis, scale validation, cross-cultural studies, and others.

Specifications Table

**Table:** 

Subject	Social Sciences
Specific subject area	Subjective well-being, happiness.
Type of data	Table and Figure.
Data collection	Data were collected using The Oxford Happiness Questionnaire (OHQ) by Hills and Argyle (2002), which consists of 29 items with response options on a 1–5 Likert scale (1=strongly disagree to 5 = strongly agree) from 804 university students in Indonesia.
Data source location	The study was conducted in Indonesia, with respondents located across several provinces, including East Java, Central Java, Yogyakarta, Kalimantan, Sulawesi, Sumatra (Aceh), Nusa Tenggara (West and East), Maluku, and Bali, https://maps.app.goo.gl/pVSfVD6QUB5XYjEm6
Data accessibility	Repository name: Mendeley Data Data identification number: DOI: 10.17632/874g7dnb99.5 Direct URL to data: https://data.mendeley.com/datasets/874g7dnb99/5 Instructions for accessing these data: The data can be accessed via the provided link. No special permissions are required; anyone with the link can view and download the dataset during the review process.
Related research article	Efendy, M., Pratitis, N., Rhivent, N. M., & Nur, A. P. E. (2024). Coping religius dan kesejahteraan psikologis mahasiswa Muslim di Indonesia. Jurnal Psikologi: Jurnal Ilmiah Fakultas Psikologi Universitas Yudharta Pasuruan, 11(2), 223–241. https://doi.org/10.35891/jip.v11i2.5506.

## 1 Value of the Data

• This dataset provides the Indonesian version of the Oxford Happiness Questionnaire (OHQ) that has been adapted across cultures through translation, back-translation, and analysis by experts, thereby expanding the use of OHQ for research purposes, and subjective well-being assessments in Indonesian-speaking students.
• This dataset contains item-level responses from 804 students in Indonesia equipped with demographic information such as gender, age, education level, and province of origin. This information is useful for researchers in exploring subjective well-being differences based on demographic groups, as well as compiling normative data for the student population in Indonesia.
• This dataset can be reused by researchers in the fields of psychology, education, public health, sociology, behavioral science, and applied statistics for various analysis purposes such as psychometric evaluation, Confirmatory Factor Analysis (CFA), measurement invariance testing, latent profile analysis, Item Response Theory (IRT), cross-cultural comparisons, and application of machine learning.
• This dataset supports evidence-based decision-making by university administrators, mental health professionals, counselors, governments, and policymakers. This data can be used to identify student groups with lower subjective levels of well-being, monitor student well-being conditions, evaluate the effectiveness of intervention programs, and design mental health promotion policies and programs aimed at improving the psychological well-being and quality of life of students in Indonesia.

## 2 Background

This dataset contains a collection of data on subjective well-being (happiness) among 804 university students in Indonesia, collected using a standardized scale—the Oxford Happiness Questionnaire (OHQ) from [1] which consists of 29 Likert-type items. This study was based on the initial objective of examining the state of subjective well-being (happiness) among university students in Indonesia, given the prevalence of incidents indicating low levels of subjective well-being among students in the country. Subjective well-being defines happiness as an individual’s positive evaluation of their life, encompassing both affective and cognitive dimensions [1].

## 3 Data Description

The respondents in this study consisted of 804 Indonesian university students, including 276 men (34.3%) and 528 women (65.7%); the mean age was 21.23 years, with a standard deviation of 3.73. The instrument used was the Oxford Happiness Questionnaire, which consists of 29 items measured on a 5-point Likert scale.

First, descriptive analysis was conducted on 29 items to determine their mean and standard deviation. Additionally, a confirmatory factor analysis (CFA) was performed to validate the constructs and calculate factor loadings, CR, and AVE. Internal consistency reliability was assessed using Cronbach’s alpha and McDonald’s omega. The results of the analysis are presented in Table 1.

**Table 1:** Descriptive statistics and psychometric properties of the full 29-item OHQ.

No	Items	Mean	SD	Loading Factor	Cronbach’s Alpha	McDonald's Omega	CR	AVE
1	I don’t feel particularly pleased with the way I am (Saya tidak senang dengan kondisi saya saat ini.)	3.12	1.144	0.466	0.888	0.893	0.892	0.307
2	I am intensely interested in other people (Saya sangat tertarik pada orang lain)	3.45	0.923	0.199				
3	I feel that life is very rewarding (Saya merasa hidup ini sangat bermanfaat)	4.01	0.861	0.620				
4	I have very warm feelings towards almost everyone (Saya mempunyai perasaan yang hangat hampir terhadap semua orang)	3.45	0.951	0.421				
5	I rarely wake up feeling rested (Saya jarang bangun pagi dengan perasaan nyaman)	3.22	1.111	0.486				
6	I am not particularly optimistic about the future (Saya tidak terlalu optimis dengan masa depan saya)	3.35	1.186	0.503				
7	I find most things amusing (Menurut saya banyak hal lucu dalam hidup ini)	4.05	0.833	0.131				
8	I am always committed and involved (Saya selalu berkomitmen dan terlibat dalam setiap kegiatan bermanfaat)	3.76	0.858	0.515				
9	Life is good (Menurut saya, hidup itu baik)	4.10	0.901	0.661				
10	I do not think that the world is a good place (Menurut saya dunia ini bukanlah tempat yang baik)	3.44	1.137	0.505				
11	I laugh a lot (Saya lebih banyak tertawa)	3.51	0.928	0.373				
12	I am well satisfied about everything in my life (Saya merasa puas dengan segala hal dalam hidup saya)	3.38	0.987	0.602				
13	I don’t think I look attractive (Menurut saya, penampilan saya kurang menarik)	2.96	1.164	0.465				
14	There is a gap between what I would like to do and what I have done (Ada kesenjangan antara apa yang ingin saya lakukan dan apa yang telah saya lakukan)	2.52	0.975	0.409				
15	I am very happy (Saya sangat senang)	3.58	0.988	0.705				
16	I find beauty in some things (Saya menemukan keindahan dalam beberapa hal dalam hidup ini)	4.08	0.769	0.631				
17	I always have a cheerful effect on others (Saya selalu memberikan kesan ceria pada orang lain)	3.79	0.936	0.475				
18	I can fit in everything I want to (Saya bisa menyesuaikan diri)	3.88	0.885	0.544				
19	I feel that I am not especially in control of my life (Saya merasa bahwa saya tidak punya kendali atas hidup saya)	3.28	1.173	0.439				
20	I feel able to take anything on (Saya merasa mampu melakukan apa pun yang saya mau)	3.45	0.972	0.527				
21	I feel fully mentally alert (Saya merasa waspada secara mental)	3.66	0.936	−0.108				
22	I often experience joy and elation (Saya sering mengalami kegembiraan dan kegembiraan)	3.54	0.900	0.585				
23	I do not find it easy to make decisions (Saya merasa tidak mudah dalam mengambil keputusan)	2.56	1.083	0.410				
24	I do not have a particular sense of meaning and purpose in my life (Saya tidak mempunyai arti dan tujuan tertentu dalam hidup saya)	3.49	1.184	0.551				
25	I feel I have a great deal of energy (Saya merasa mempunyai banyak energi)	3.34	1.032	0.633				
26	I usually have a good influence on events (Saya biasanya mempunyai pengaruh yang baik pada suatu peristiwa)	3.61	0.830	0.502				
27	I do not have fun with other people (Saya tidak bersenang-senang dengan orang lain)	3.39	0.974	0.355				
28	I don’t feel particularly healthy (Saya merasa tidak sehat)	3.37	1.104	0.570				
29	I do not have particularly happy memories of the past (Saya tidak memiliki kenangan indah tentang masa lalu)	3.33	1.246	0.300				

Next, items that did not meet the factor loading threshold were eliminated; only items with a factor loadings >0.40 were selected, as suggested in [3], where factor loadings of 0.4–0.7 can be considered for retention. Therefore, Item 2, Item 7, Item 11, Item 21, Item 27, and Item 29 were removed from the measurement model, leaving 23 items that met the factor loading threshold of >0.4.

This dataset presents two sets of measurement results. The first, in Table 1, consists of measurements for the entire 29-item scale (descriptive statistics, factor loadings, CR, and AVE). The second, in Table 2, contains measurement results for the 23-item scale, with recalculated descriptive statistics, factor loadings, CR, and AVE. All data files are available in the Mendeley Data repository (DOI:10.17632/874 *g*7dnb99.5), along with raw data, processed datasets, descriptive statistics output, reliability output, and factor analysis output. This dataset is designed to allow direct access to both the original and simplified measurement models.

**Table 2:** Descriptive statistics and psychometric properties of the reduced 23-item OHQ.

No	Items	Mean	SD	Loading Factor	Cronbach’s alpha	McDonald's Omega	CR	AVE
1	I don’t feel particularly pleased with the way I am (Saya tidak senang dengan kondisi saya saat ini)	3.12	1.144	0.461	0.899	0.902	0.901	0.288
3	I feel that life is very rewarding (Saya merasa hidup ini sangat bermanfaat)	4.01	0.861	0.625				
4	I have very warm feelings towards almost everyone (Saya mempunyai perasaan yang hangat hampir terhadap semua orang)	3.45	0.951	0.416				
5	I rarely wake up feeling rested (Saya jarang bangun pagi dengan perasaan nyaman)	3.22	1.111	0.486				
6	I am not particularly optimistic about the future (Saya tidak terlalu optimis dengan masa depan saya)	3.35	1.186	0.497				
8	I am always committed and involved (Saya selalu berkomitmen dan terlibat dalam setiap kegiatan bermanfaat)	3.76	0.858	0.521				
9	Life is good (Menurut saya, hidup itu baik)	4.10	0.901	0.663				
10	I do not think that the world is a good place (Menurut saya dunia ini bukanlah tempat yang baik)	3.44	1.137	0.497				
12	I am well satisfied about everything in my life (Saya merasa puas dengan segala hal dalam hidup saya)	3.38	0.987	0.610				
13	I don’t think I look attractive (Menurut saya, penampilan saya kurang menarik)	2.96	1.164	0.461				
14	There is a gap between what I would like to do and what I have done (Ada kesenjangan antara apa yang ingin saya lakukan dan apa yang telah saya lakukan)	2.52	0.975	0.410				
15	I am very happy (Saya sangat senang)	3.58	0.988	0.713				
16	I find beauty in some things (Saya menemukan keindahan dalam beberapa hal dalam hidup ini)	4.08	0.769	0.630				
17	I always have a cheerful effect on others (Saya selalu memberikan kesan ceria pada orang lain)	3.79	0.936	0.476				
18	I can fit in everything I want to (Saya bisa menyesuaikan diri)	3.88	0.885	0.551				
19	I feel that I am not especially in control of my life (Saya merasa bahwa saya tidak punya kendali atas hidup saya)	3.28	1.173	0.428				
20	I feel able to take anything on (Saya merasa mampu melakukan apa pun yang saya mau)	3.45	0.972	0.535				
22	I often experience joy and elation (Saya sering mengalami kegembiraan dan kegembiraan)	3.54	0.900	0.586				
23	I do not find it easy to make decisions (Saya merasa tidak mudah dalam mengambil keputusan)	2.56	1.083	0.401				
24	I do not have a particular sense of meaning and purpose in my life (Saya tidak mempunyai arti dan tujuan tertentu dalam hidup saya)	3.49	1.184	0.541				
25	I feel I have a great deal of energy (Saya merasa mempunyai banyak energi)	3.34	1.032	0.637				
26	I usually have a good influence on events (Saya biasanya mempunyai pengaruh yang baik pada suatu peristiwa)	3.61	0.830	0.506				
28	I don’t feel particularly healthy (Saya merasa tidak sehat)	3.12	1.144	0.461				

## 4 Experimental Design, Materials and Methods

This study employed a non-experimental design using a questionnaire, which was subsequently analyzed descriptively. Descriptive data analysis was conducted to obtain an overview of the level of happiness among university students in Indonesia. The study sample consisted of 804 students, including 276 male students (34.3%) and 528 female students (65.7%). The average age of the participants was 21.23 years (SD = 3.73). To optimize geographic coverage, respondents came from various regions in Indonesia, such as Java, Sumatra, Kalimantan, Sulawesi, Bali, Nusa Tenggara, Maluku, and others.

Participants were university students in Indonesia who are recruited using non-random convenience sampling techniques. The questionnaire link was disseminated online through WhatsApp groups, Instagram, and academic networks involving students, lecturers, and faculty colleagues who helped distribute the survey to participants who met the criteria.

The criteria for respondents in this study include: (1) being an active student, (2) being at least 18 years old, and (3) willing to participate voluntarily by filling out an informed consent sheet. All participation was voluntary, and no compensation in the form of money or materials is given to participants.

The target sample size was determined by considering the need for psychometric analysis and confirmatory factor analysis (CFA). Given that the Oxford Happiness Questionnaire (OHQ) consisted of 29 items, the final sample of 804 participants resulted in a respondent-to-item ratio of around 27.7:1. Therefore, this sample size is considered adequate for descriptive analysis, reliability testing, and confirmatory factor analysis (CFA).

The instrument used was the Oxford Happiness Questionnaire (OHQ) from [1], which consists of 29 items with a Likert scale ranging from 1 (strongly disagree) to 5 (strongly agree). The OHQ contains both favorable and unfavorable items. The favorable items were Items 2, 3, 4, 7, 8, 9, 11, 12, 15, 16, 17, 18, 20, 21, 22, 25, and 26. The unfavorable items requiring reverse coding were Items 1, 5, 6, 10, 13, 14, 19, 23, 24, 27, 28, and 29. The scale used has undergone an adaptation process following the procedures in [2], starting with translation by a language expert, back-translation, synthesis, item review by experts to ensure conceptual equivalence, and Confirmatory Factor Analysis.

All data were analyzed statistically using Jamovi software (version 1.6.23.0). The specific analyses conducted included descriptive analysis, reliability analysis, confirmatory factor analysis (CFA), and item reduction procedures in accordance with the recommended thresholds; items with factor loadings < 0.40 were eliminated, namely Item 2, Item 7, Item 11, Item 21, Item 27, and Item 29; and the remaining 23 items were then reanalyzed for descriptive statistics, factor loadings, composite reliability, and average variance extracted.

All data files (raw data, processed datasets, descriptive statistics, reliability result, and CFA results) can be found in the Mendeley Data Repository (DOI: 10.17632/874g7dnb99.5)

## Limitations

The limitations of this study include the use of non-random sampling; respondents completed the questionnaire based on self-assessment, which may have introduced bias; and the proportion of respondents from each province was not evenly distributed

## Ethics Statement

Since this study involved human participants, it was conducted in accordance with the Helsinki Principles [4]. All participants in this study provided their consent before completing the questionnaire, meaning their participation was voluntary and free from coercion. Data were collected via an online questionnaire and did not include any personally identifiable information, thereby ensuring the confidentiality and anonymity of all participants. This study was approved by the Institutional Ethics Committee of the University of 17 Agustus 1945 Surabaya

## CRediT Author Statement

**Mamang Efendy:** Conceptualizing; Developing a scale; Conducting descriptive and item analyses; Writing articles; Supervising research; **Andik Matulessy:** Ensuring the accuracy of the research methodology; Conducting validity analyses; Writing and reviewing the manuscript; **Amanda Pasca Rini:** Performing data administration and scoring; assisting with data collection and analysis; assisting with the writing of articles and reviews; **Diah Sofiah:** Assisting with the administration of reliability test data analysis; distributing research questionnaires; monitoring the number of respondents who completed the questionnaires and ensuring provincial representation; assisting with article editing.

## Data Availability

Mendeley DataSubjective well-being (happiness) data among Indonesian university students (Original data).

## References

[1] Hills P., Argyle M. (2002). The Oxford Happiness Questionnaire: a compact scale for the measurement of psychological well-being. Pers. Individ. Dif..

[2] Beaton D.E., Bombardier C., Guillemin F., Ferraz M.B. (2000). Guidelines for the process of cross-cultural adaptation of self-report measures. Spine (Phila. Pa. 1976)..

[3] J.F. Hair, W.C. Black, B.J. Babin, and R.E. Anderson, “Multivariate data analysis,” 2019.

[4] Association W.M. (2013). World Medical Association Declaration of Helsinki: ethical principles for medical research involving human subjects. Jama.

